# Supplementary material for: The interactome of intact mitochondria by cross-linking mass spectrometry provides evidence for coexisting respiratory supercomplexes
Source: Mol Cell Proteomics. 2017 Dec 8;17(2):216–32. doi: 10.1074/mcp.RA117.000470 (PMC5795388; doi:10.1074/mcp.RA117.000470)
Supplement: Supplemental Data [file supp_RA117.000470_133922_0_supp_23978_fzffwf.zip › spectra_annotation/mito_spectra_annotation/198-1-14-1-6-1.pdf]

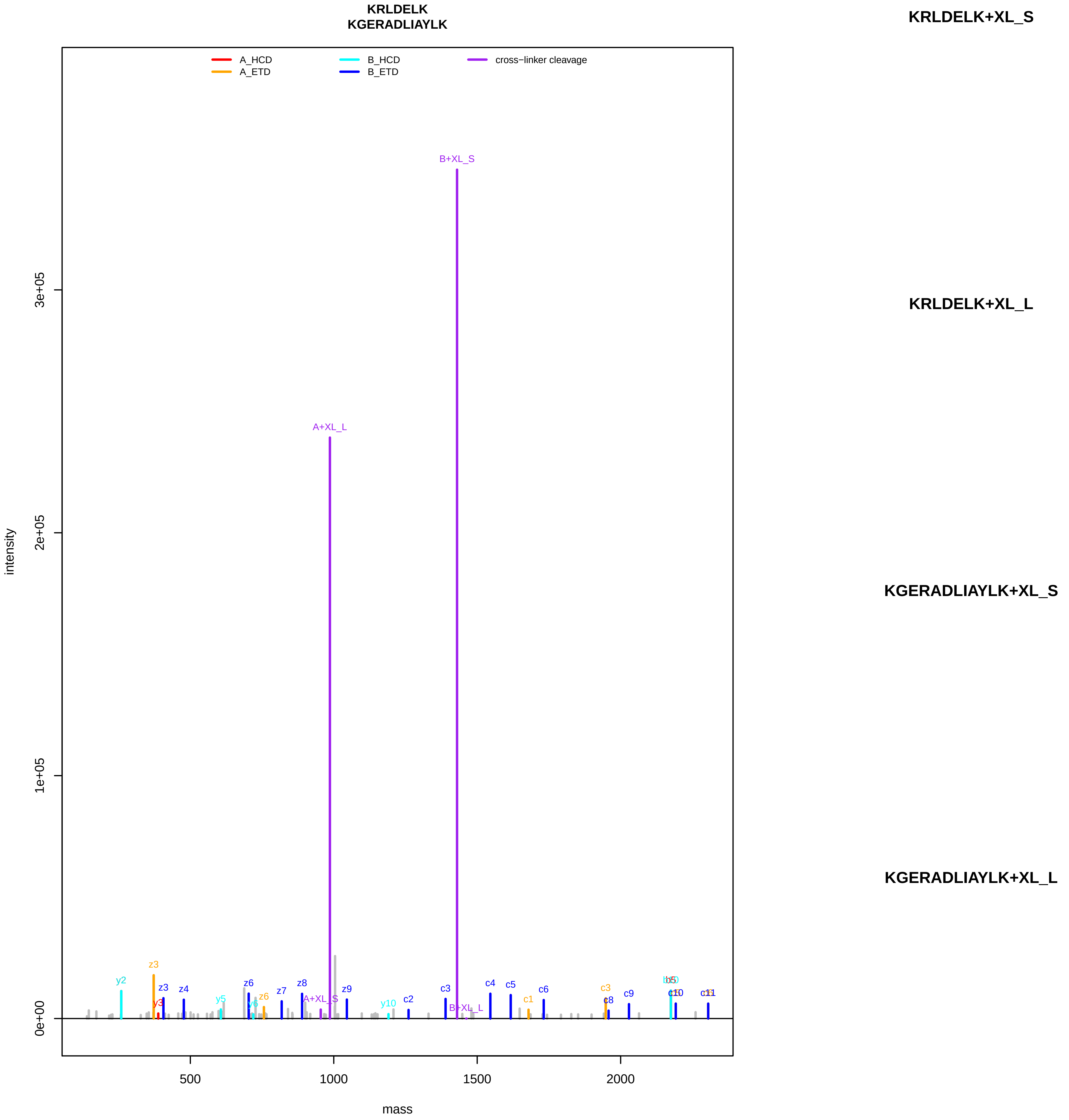
KRLDELK
KGERADLIAYLK
KRLDELK+XL_S
KRLDELK+XL_L
KGERADLIAYLK+XL_S
KGERADLIAYLK+XL_L
A_HCD
A_ETD
B_HCD
B_ETD
cross−linker cleavage
B+XL_S
A+XL_L
A+XL_S
B+XL_L
intensity
mass
0e+00
1e+05
2e+05
3e+05
500
1000
1500
2000
y2
z3
y3
z4
y5
z6
y6
z7
z8
z9
y10
c2
c3
c4
c5
c1
c6
c8
c9
c10
c11
